# Supplementary material for: A Cryptochrome adopts distinct moon- and sunlight states and functions as sun- versus moonlight interpreter in monthly oscillator entrainment
Source: Nat Commun. 2022 Sep 5;13:5220. doi: 10.1038/s41467-022-32562-z (PMC9445029; doi:10.1038/s41467-022-32562-z)
Supplement: Supplementary file 1 — Supplementary Information [file 41467_2022_32562_MOESM1_ESM.pdf]

Supplementary Information for

A Cryptochrome adopts distinct moon- and sunlight states and functions as sun- versus moonlight interpreter in monthly oscillator entrainment

Birgit Poehn*[1,2], Shruthi Krishnan*[3,4], Martin Zurl[1,2], Aida Coric#[1,2], Dunja Rokvic#[1,2], N. Sören Häfker[1,2], Elmar Jaenicke[3], Enrique Arboleda[1,2,5], Lukas Orel[1,2], Florian Raible[1,2], Eva Wolf@[3,4] and Kristin Tessmar-Raible@[1,2,6,7]

Supplementary Figures

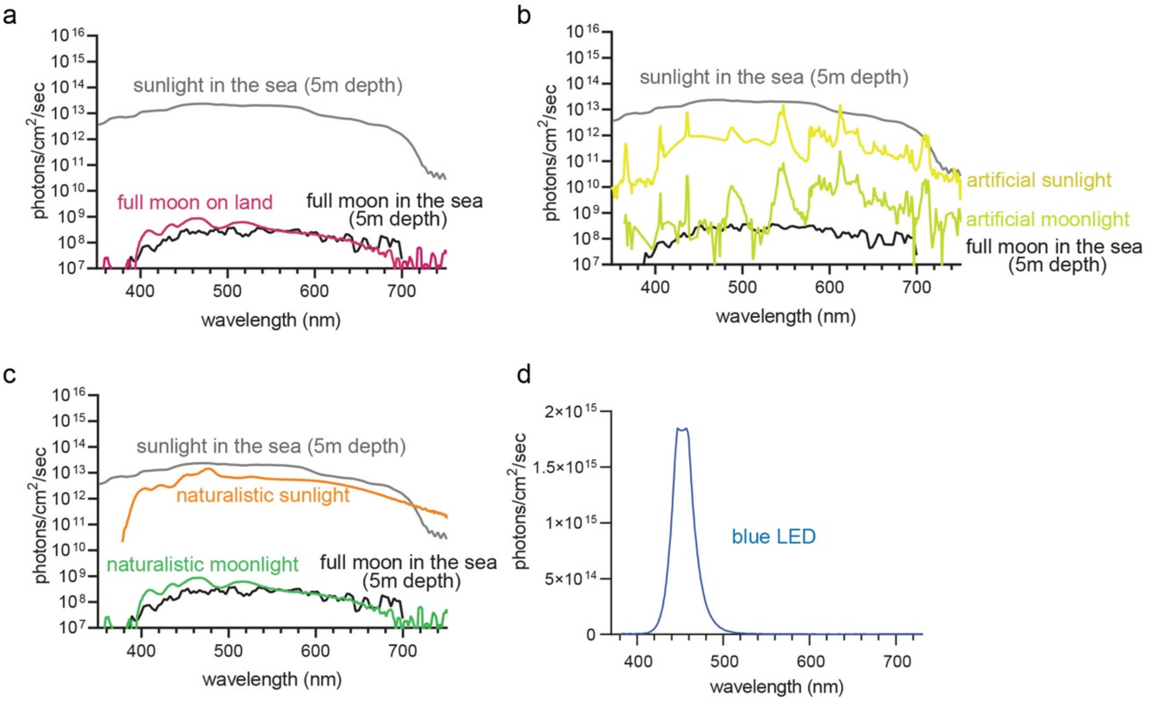

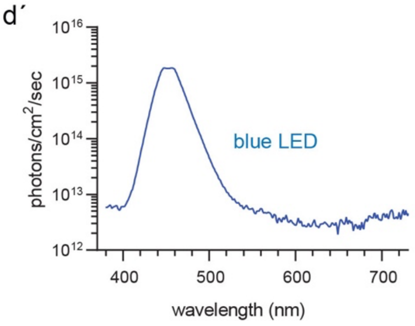

e

	photons/cm2/s		
**light source**	**[380nm-750nm]**	**[330nm-750nm]**	**[330nm-510nm]**
full moon in the sea (5m depth)	$5.8x10^{10}$	$5.8x10^{10}$	$2.3x10^{10}$
full moon on land	$1.8x10^{11}$	$1.8x10^{11}$	$3.7x10^{10}$
artificial moonlight	$2.5x10^{12}$	$2.5x10^{12}$	$1.9x10^{12}$
naturalistic moonlight	$9.7x10^{10}$	$9.7x10^{10}$	$5.4x10^{10}$
sunlight in the sea (5m depth)	$4.4x10^{15}$	$4.6x10^{15}$	$2.5x10^{15}$
artificial sunlight	$3.2x10^{14}$	$3.2x10^{14}$	$1.0x10^{14}$
naturalistic sunlight	$1.6x10^{15}$	$1.6x10^{15}$	$7.5x10^{14}$
blue LED	$6.2x10^{16}$		

Supplementary Figure 1: Spectra of light sources

(**a**) Light spectra in nature: sunlight (grey) and full moon in 5m depth in Ischia (black) as well as full-moon light on land (red). **(b)** Spectra of the highly artificial sun- (yellow) and moonlight (light green) in the worm culture room. **(c)** Spectra of designed naturalistic sun- (orange) and moonlight (green). **(d, d')** Spectrum of LED used for spectroscopic experiments with a blue-light dominated spectrum. d) linear, d') logarithmic plot. Also see Suppl.Mat.1 for details provided by company. **(e)** Summed number of photons/cm^2/s of (a-d) for different spectral ranges. 330- 510 nm corresponds to the main absorbance range of the FAD cofactor. Intensity and spectrum were always measured in the distance relevant for the experiments.

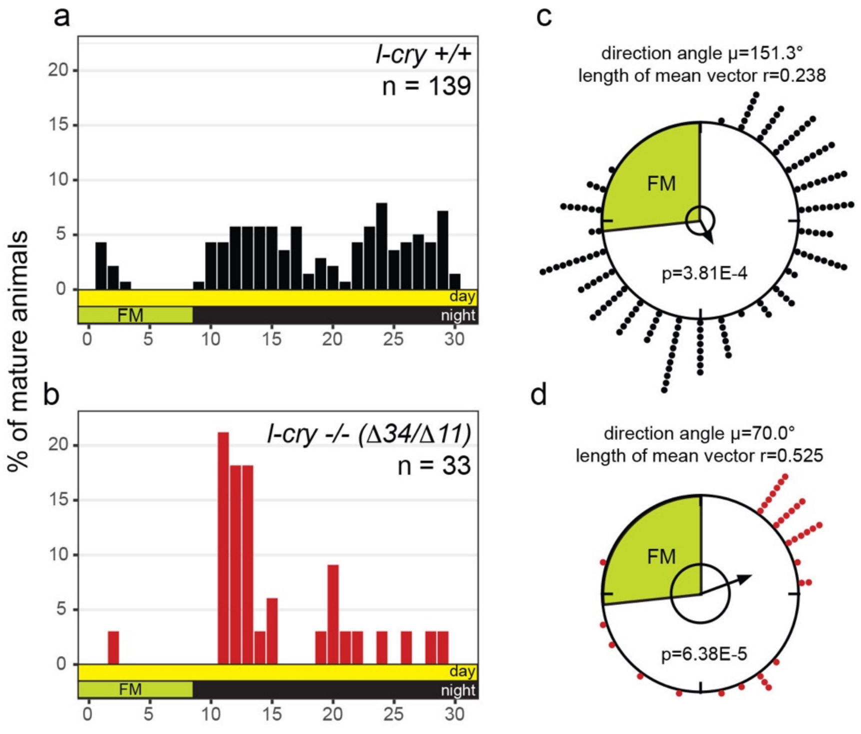

e

Comparison	Kolmogorov-Smirnov test (p)	Mardia-Watson-Wheeler test (p)	phase difference of μ (°)	phase difference of vectors (days)
l-cry +/+ vs *l-cry -/- (Δ11/Δ34)*	0.007	$8.24 \cdot 10^{-4}$	81.3	6.8
Sea vs *l-cry -/- (Δ11/Δ34)*	0.207	0.635	13.5	1.1
Sea vs *l-cry* +/+	$4 \cdot 10^{-15}$	$<10^{-12}$	94.8	7.9

Supplementary Figure 2: *l-cry* transheterozygous (Δ11/Δ34) mutants show increased spawning synchrony

(a-b) Spawning of *l-cry +/+* (a) and *l-cry -/-(Δ11/Δ34)* (b) animals over the lunar month under 8 nights of standard worm room culture full moon. **(c,d)** Same data as in (a,b) plotted as circular data. 360° correspond to 30 days of the lunar month. The arrow represents the mean vector characterized by the direction angle μ and r. r (length of μ) indicates phase coherence (measure of population synchrony). p-values inside the plots are results of Rayleigh Tests: Significance indicates non-random distribution of data points. The inner circle represents the Rayleigh critical value (p=0.05). **(e)** Results of two-sided multi-sample statistics on spawning data shown in (a-d). Phase differences in days can be calculated from the angle between the two mean vectors (i.e. 12° = 1 day).

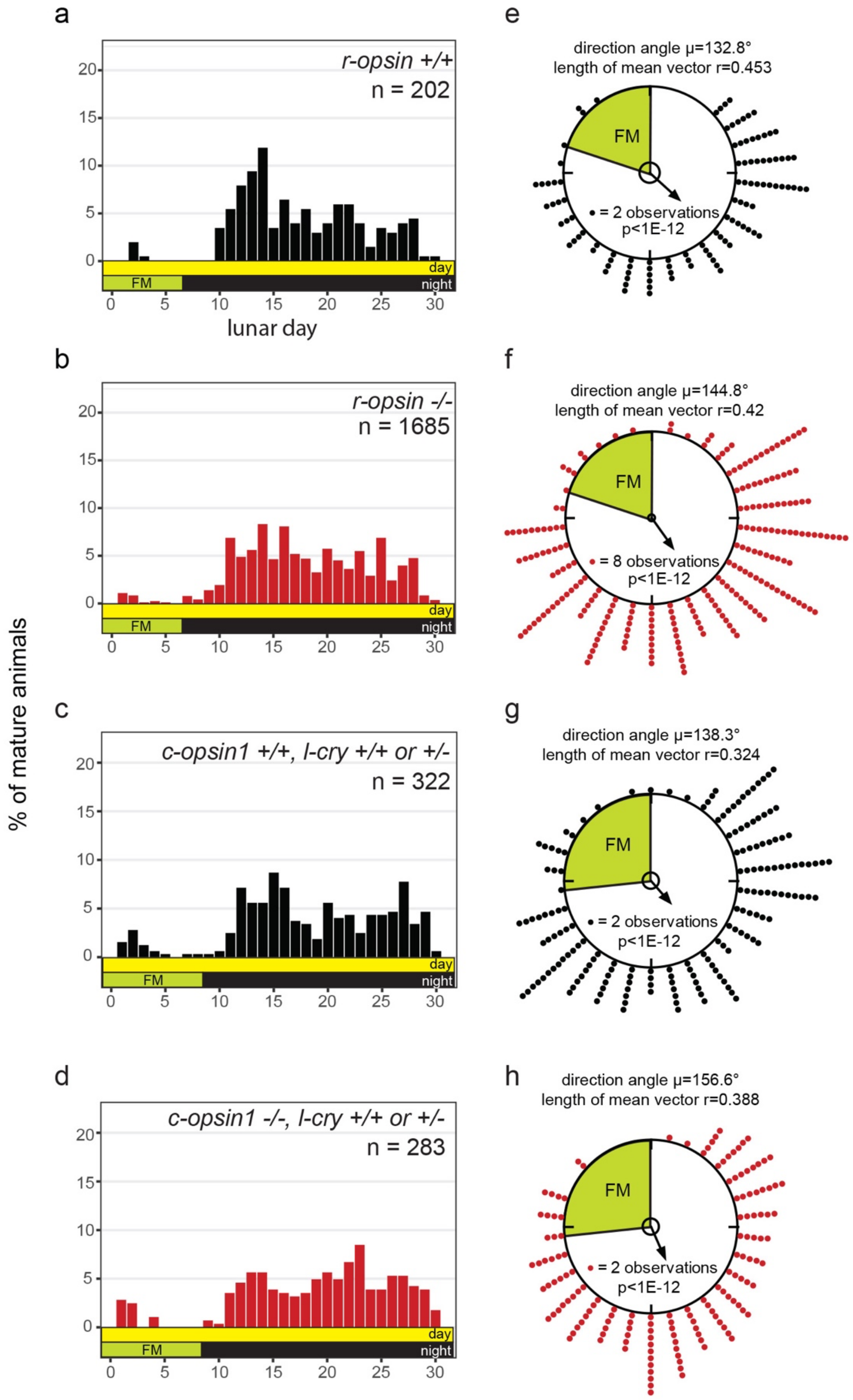

i

Comparison	Kolmogorov-Smirnov Test (p)	Mardia-Watson-Wheeler Test (p)	phase difference of μ (°)	phase difference of vectors (days)
r-opsin +/+ vs -/-	0.204	0.263	11.9	1.0
c-opsin1 +/+ vs -/-	0.053	0.069	18.3	1.5

Supplementary Figure 3: *r-opsin* and *c-opsin1* mutants show no alterations in spawning synchrony

(a-d) Spawning of indicated genotypes under standard laboratory conditions with 6 nights (*r-opsin*) or 8 nights (*c-opsin1*) of full moon. Animals in c and d are *l-cry +/+* or *+/-.* **(e-h)** Same data as in (a-d) plotted as circular data. 360° correspond to 30 days of the lunar month. The arrow represents the mean vector characterized by the direction angle µ and r. r (length of µ) indicates phase coherence (measure of population synchrony). p-values inside the plots are results of Rayleigh Tests: Significance indicates non-random distribution of data points. The inner circle represents the Rayleigh critical value ($p=0.05$). **(i)** Results of two-sample multi-sample statistics on spawning data shown in (a-h). Phase differences in days can be calculated from the angle between the two mean vectors (i.e. 12° = 1 day).

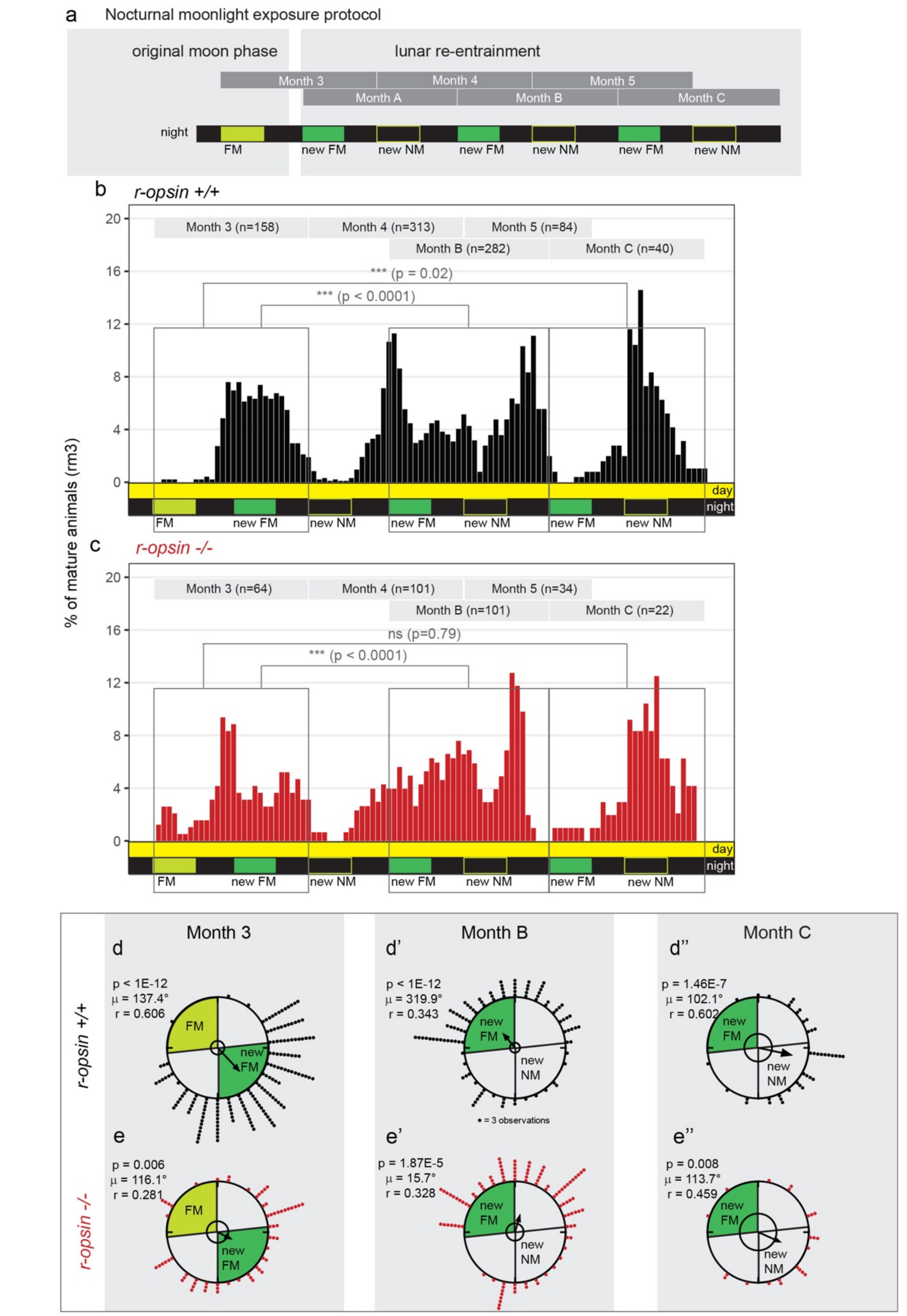

f *r-opsin +/+*

Comparison	Kolmogorov-Smirnov Test (p)	Mardia-Watson-Wheeler Test (p)	phase difference of vectors (days)
Month 3 vs B	< 2.2E-16	< 1E-12	14.8
Month 3 vs C	0.02	0.11	2.9

g *r-opsin -/-*

Comparison	Kolmogorov-Smirnov Test (p)	Mardia-Watson-Wheeler Test (p)	phase difference of vectors (days)
Month 3 vs B	2.34E-6	1.37E-4	8.4
Month 3 vs C	0.79	0.51	0.2

Supplementary Figure 4: The photoreceptor molecule *r-opsin1* does not contribute to circalunar clock entrainment. **(a)** Nocturnal moonlight exposure protocol of lunar phase shift with 8 nights of naturalistic moonlight (dark green). **(b,c)** Number of mature animals (percent per month, rolling mean with a window of 3 days) of r-opsin wildtype **(b)** and mutant **(c)** animals. p-values: two-sided Kolomogorov-Smirnov tests. **(b)** month 3 vs B: p<0.0001, month 3 vs C: p=0.01828 **(c)** month 3 vs C: p<0.0001, month 3 vs C: p=0.7928 **(d,e)** Data as in **(b,c)** plotted as circular data. 360° correspond to 30 days of the lunar month. The arrow represents the mean vector characterized by the direction angle μ and r. r (length of μ) indicates phase coherence (measure of population synchrony). p-values are results of Rayleigh Tests: Significance indicates non-random distribution of data points. The inner circle represents the Rayleigh critical value (p=0.05). **(f,g)** Results of two-sided multisample statistics on spawning data shown in **(a-e).** Phase differences in days were calculated from the angle between the two mean vectors (i.e. 12°= 1 day).

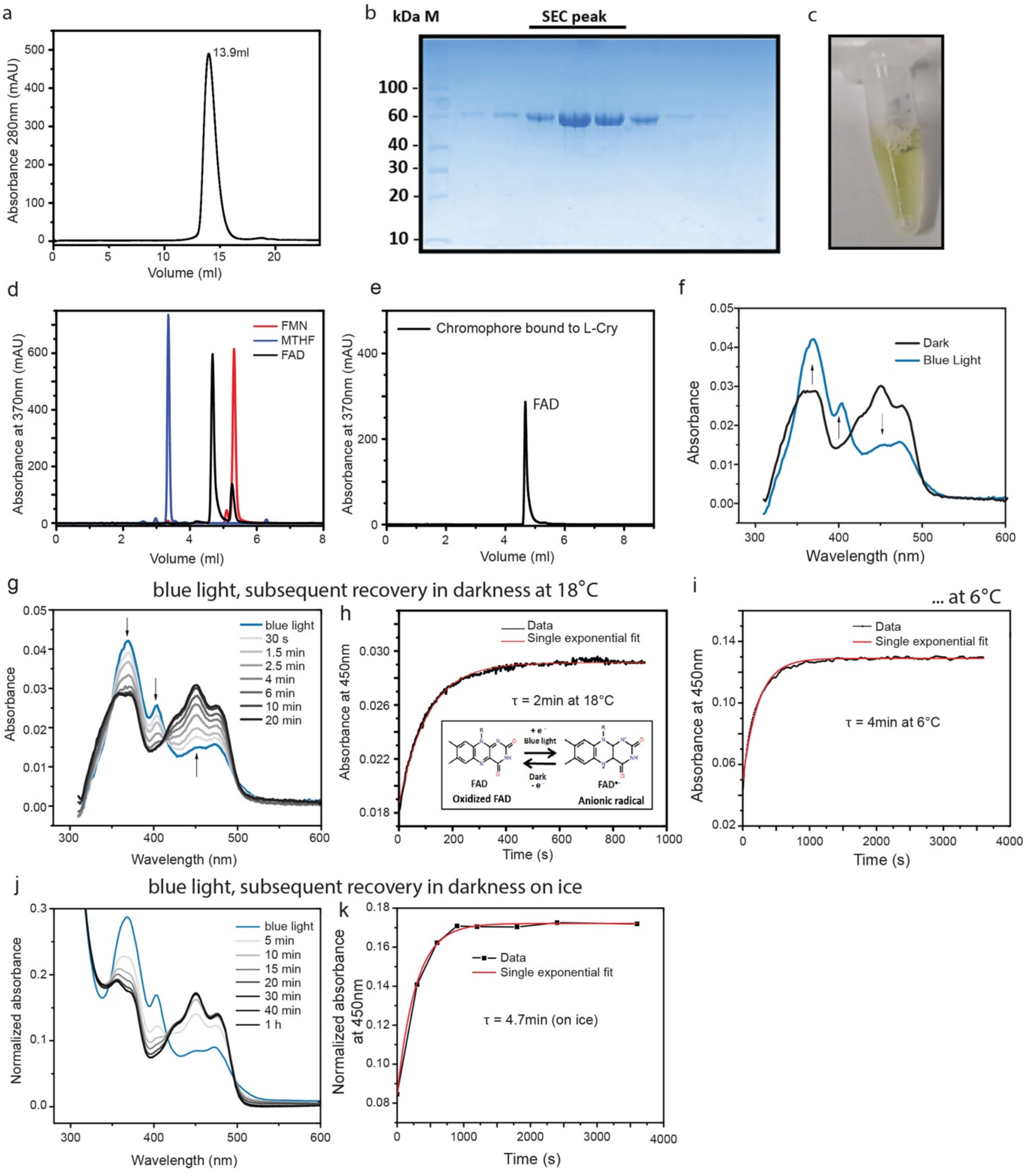

Supplementary Figure 5: Purification, biochemical and spectral characterization of L-Cry with light with a blue-light dominated spectrum (referred to as "blue light").

(a) Size-exclusion chromatography (SEC) of L-Cry on analytical S200 10/300 column. L-Cry elutes at 13.9 ml, suggesting a homodimer based on calibration standards. **(b)** 10% Bis-Tris gel loaded with fractions from the L-Cry SEC peak in (a). **(c)** L-Cry protein solution (5 mg/ml) with yellow color from bound oxidized FAD. **(d,e)** Reverse phase HPLC analysis identifies FAD as only L-Cry chromophore. Elution profile of standard chromophores FMN, MTHF and FAD (d) were compared with the L-Cry bound chromophore obtained after heat denaturation (e). **(f)** Absorption spectrum of L-Cry in dark (black) and after 110 s blue light (blue). Arrows indicate the change in absorbance at 370 nm, 404 nm and 450 nm between FAD (dark) and $FAD^{\circ -}$ (after blue light). **(g,h)** L-Cry dark recovery after blue light activation at 18 °C. Full Spectra in (g), 450 nm absorbance in (h). Inset in (h): Model of FAD photoreaction. **(i)** Dark recovery after blue light activation at 6 °C (450 nm absorbance). **(j,k)** Dark recovery after blue light activation on ice. Full spectra in J, 450 nm absorbance in k. Absorbances in were

normalised when a shift in the baseline occurred between different measurements of the same measurement set, which is then indicated on the Y-axis as "normalized absorbance".

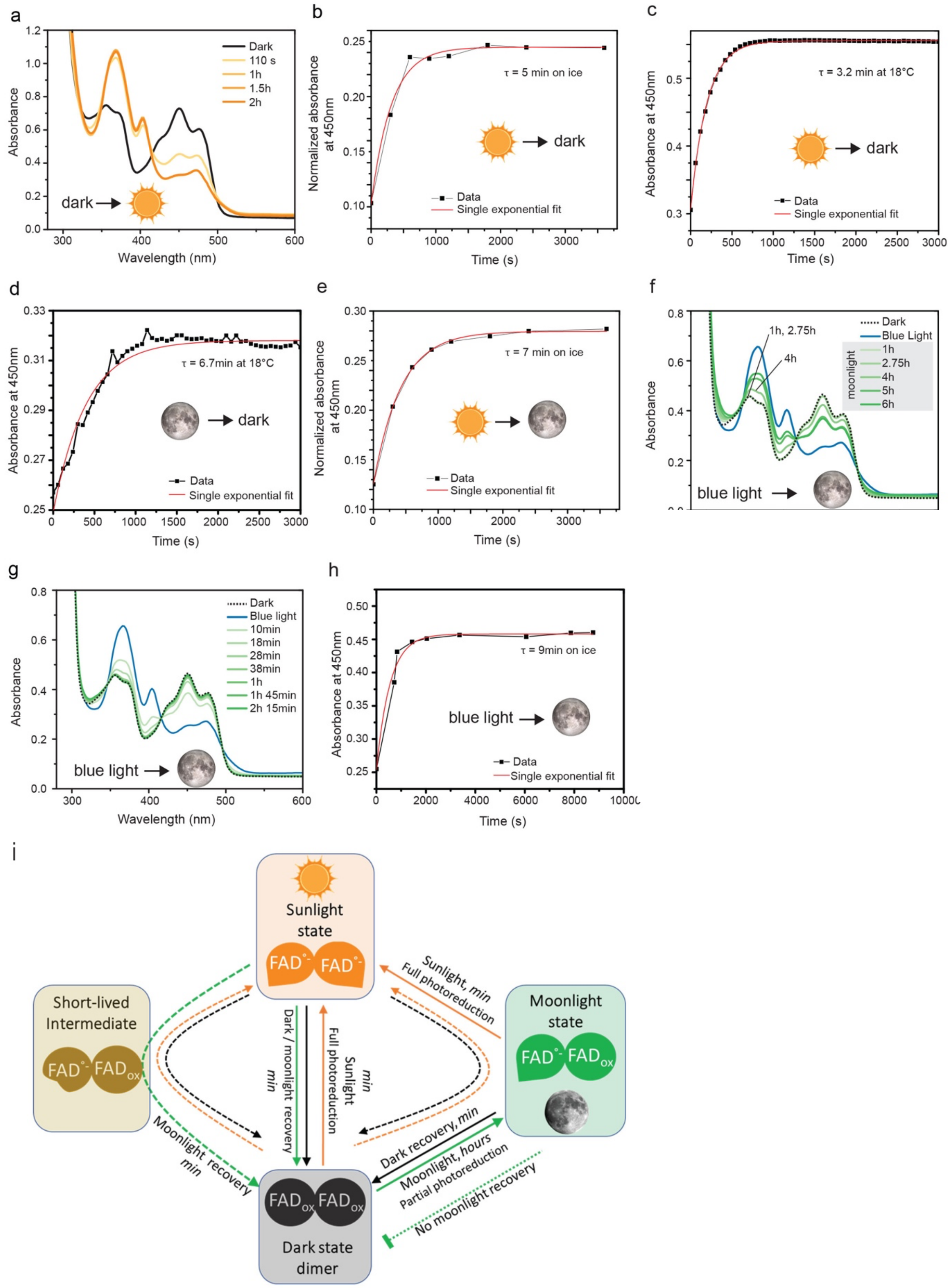

Supplementary Figure 6: Spectral characterization of L-Cry under naturalistic sun- and moonlight

(a) Absorption spectrum of L-Cry in dark (black) and after sunlight exposure (orange). Additional timepoints shown in Fig. 5b. **(b)** Dark recovery of L-Cry after 20 min of sunlight on ice: absorbance at 450 nm, full spectra in Fig. 5c. **(c)** Dark recovery of L-Cry after 20 min sunlight at 18 °C: absorbance at 450 nm. **(d)** Dark recovery of L-Cry after 6 hours of naturalistic moonlight: absorbance at 450 nm. Full spectra in Fig. 5f. **(e)** Absorbance at 450 nm after 20 min sunlight followed by dark-state recovery in presence of moonlight. Full spectra in Fig. 5h. **(f)** Absorption spectra of L-Cry after 110 s blue light illumination followed by an up to 6 h exposure to naturalistic moonlight. L-Cry first returns to the dark state (1 h, 2.45 h) and after 3 h starts to build the moonlight state (4 h, 5 h, 6 h). **(g)** Recovery of oxidized FAD from the blue light induced anionic $FAD^{\circ-}$ radical under naturalistic moonlight shows that L-Cry first returns to the dark state. **(h)** Absorbance values at 450 nm from g). Note: strong blue light results in L-Cry's sunlight state. (b,e) Absorbances in were normalised as a shift in the baseline occurred between different measurements of the same measurement set, which is indicated as "normalized absorbance". **(i)** Model of transitions between different L-Cry states. Solid arrows depict transitions between stably accumulating states as observed by UV/VIS spectroscopy (also see Fig. 5j). Our data suggest that L-Cry forms an asymmetric dimer, where the flavin cofactor of one monomer (with a high quantum yield) can be photoreduced by ~6 hours moonlight illumination, resulting in a half-reduced $FAD^{\circ-}$ FAD_{ox} moonlight state (right). Sunlight can photoreduce both L-Cry monomers within minutes, resulting in the fully photoreduced $FAD^{\circ-}$ $FAD^{\circ-}$ sunlight state. Dark state recovery of the sunlight state in presence of moonlight (dashed green arrow) can only occur via a short-lived half-reduced $FAD^{\circ-}$ FAD_{ox} intermediate (left), that is conformationally and kinetically different from the "true" moonlight state (right), because moonlight would maintain the moonlight state (green dotted line, "no moonlight recovery"). Note that the moonlight- to sunlight state transition (direct sunlight photoactivation) and the sunlight- to moonlight state transition (via the FAD_{ox} FAD_{ox} dark state) follow distinct and unidirectional pathways. Sunlight photoreduction of the FAD_{ox} FAD_{ox} dark state and dark recovery of the sunlight state could go via the moonlight state (right) or via the altered $FAD^{\circ-}FAD_{ox}$ intermediate (left) (dashed orange (sunlight) and black (darkness) arrows). For dark recovery of the sunlight state, which is not impacted by the different photoreduction quantum yields of the two L-Cry monomers, a concerted transition from $FAD^{\circ-}FAD^{\circ-}$ to $FAD_{ox}FAD_{ox}$ is also conceivable, where half-reduced $FAD^{\circ-}FAD_{ox}$ dimer intermediates play a negligible role.

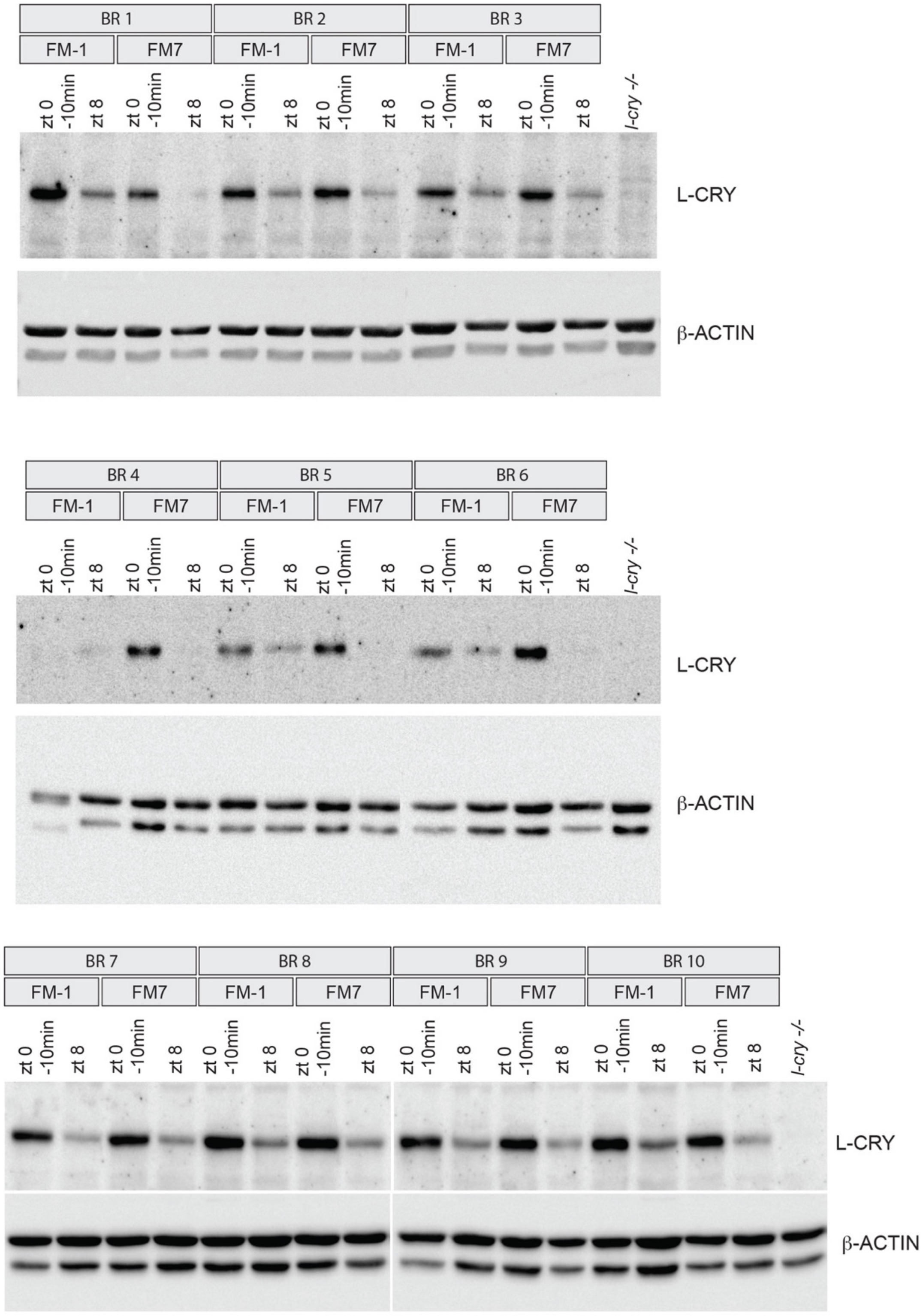

Supplementary Figure 7: Raw images of all Western blots quantified in Fig. 6b. BR: biological replicate. Note: for the upper most β-actin blot, in contrast to all other β-actin blots, goat anti-rabbit IgG StarBright™ Blue 700 antibody (BIORAD) was used as secondary antibody.

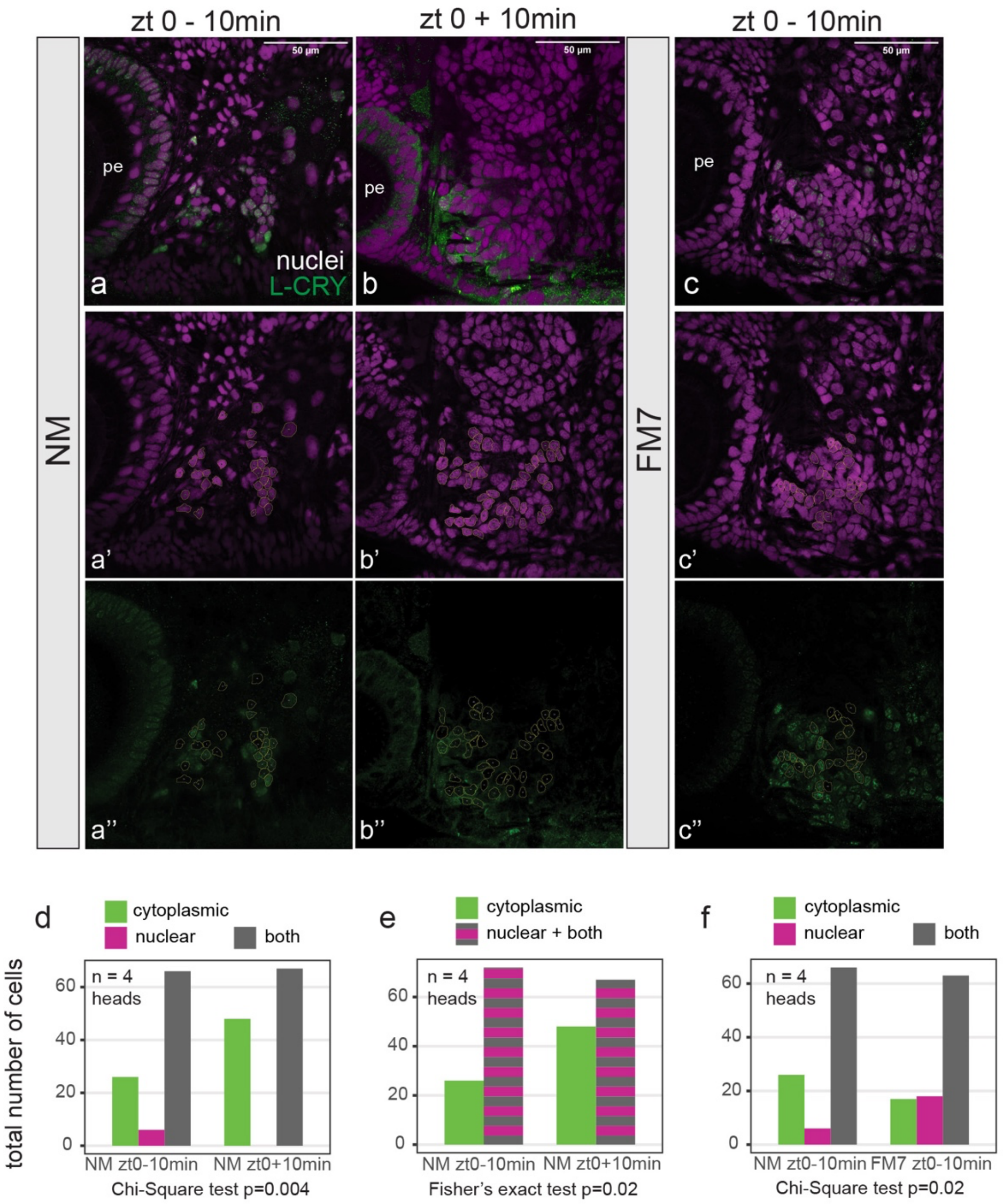

Supplementary Figure 8: L-Cry immunohistochemistry on *Pdu* heads

(a-c) Single layers of confocal images (1.28µm thick) of worm heads stained with anti-L-Cry antibody (green) and HOECHST (magenta) at indicated timepoints. Details of these pictures: Fig. 6e-g'''. pe, posterior eye. **(a'-c'')** Examples for selection of Regions of Interest (ROI) used for the quantification of L-Cry protein localization in d-f. **(d-f)** Categorical quantification of L-Cry's subcellular localization at indicated timepoints. The statistical analysis was performed according to the requirements for categorical data (24). For primary data see Suppl.Data 1.

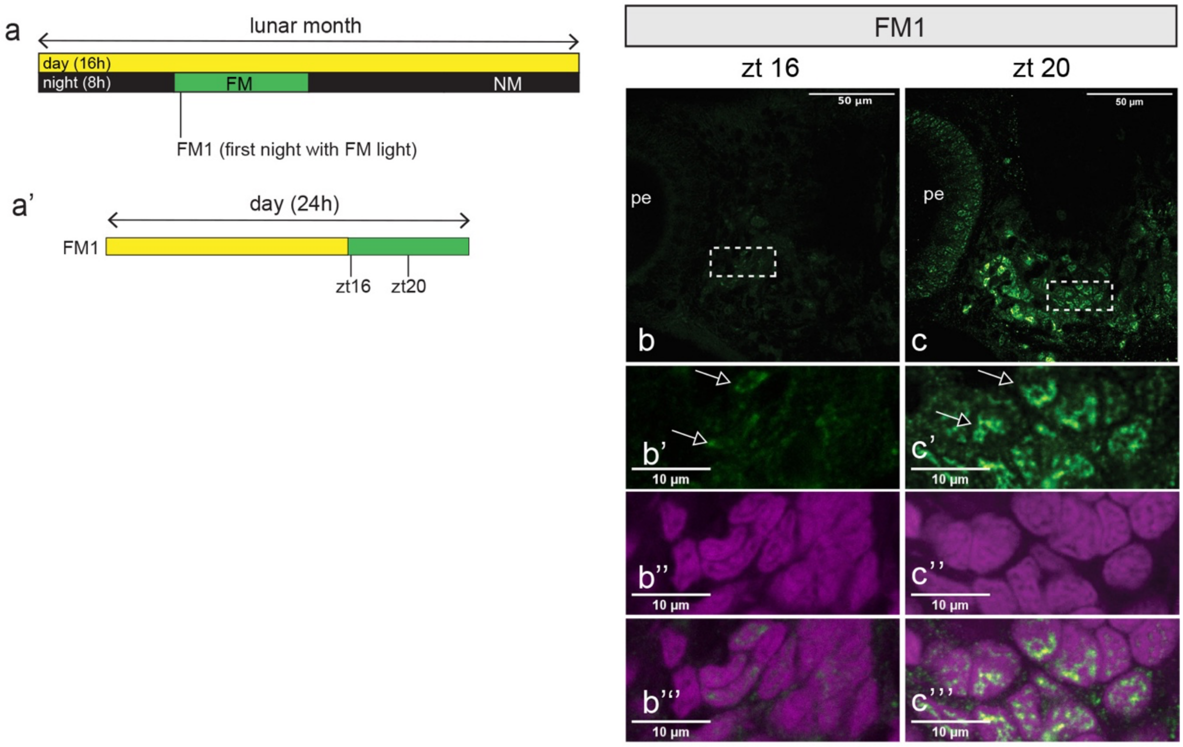

Supplementary Figure 9: L-Cry protein is endogenously present at timepoints that allow for a sufficiently long exposure to naturalistic moonlight to reach the moonlight-state

(a,a') Overview of sampling timepoints. 16hrs day (light) and 8 hrs night (dark or moonlight) per 24hrs, with 8 nights of moonlight per month. **(b-c)** Confocal single layer (1.28µm) images of worm heads stained with anti-L-Cry antibody (green). White rectangles: areas of the zoom-ins presented below. **(b'-c''')** zoomed pictures of the areas depicted in b-c. anti-L-Cry antibody (green), HOECHST (magenta: nuclei), Arrows indicate L-Cry staining. Scale bars: 10um.

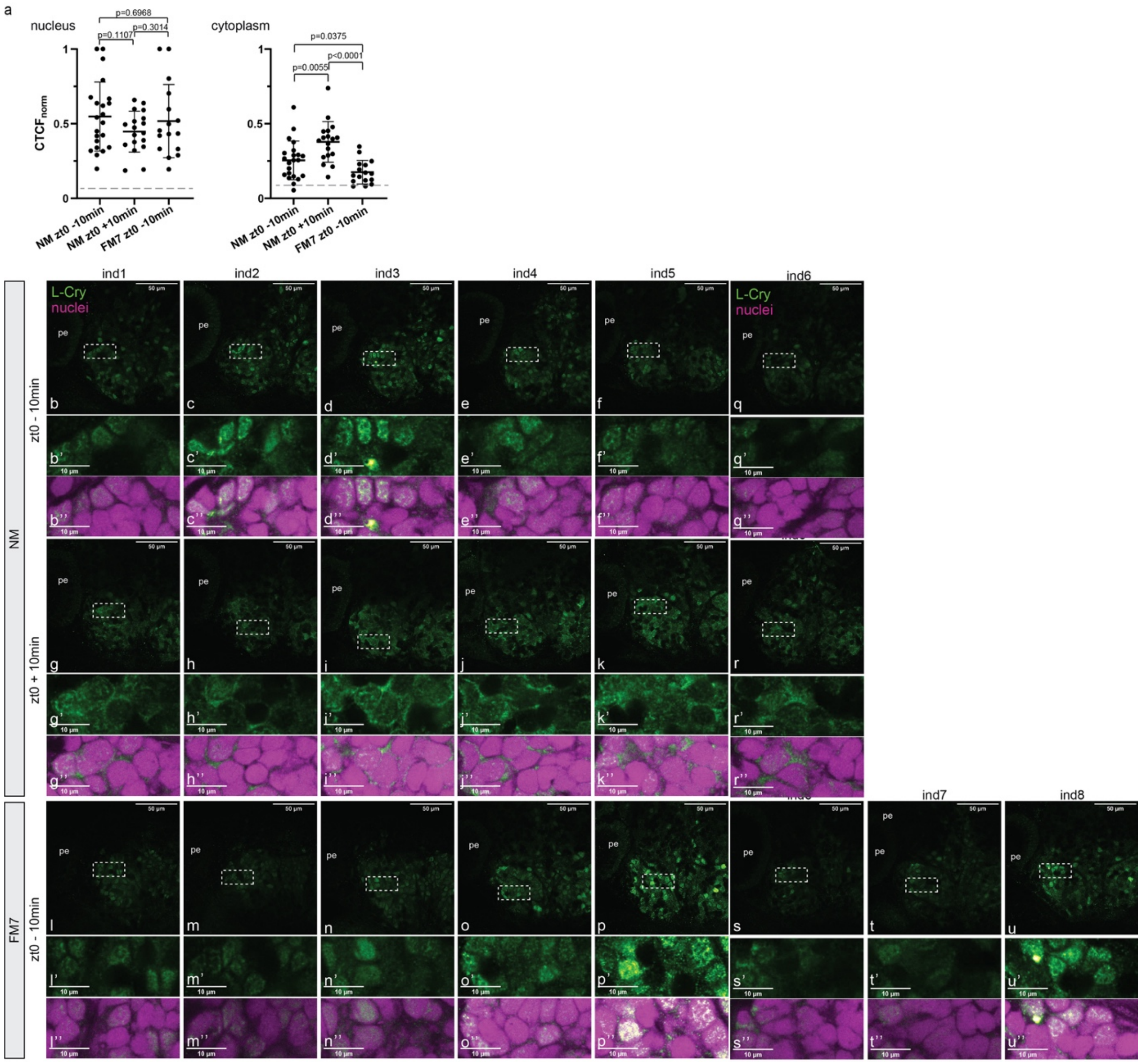

Supplementary Figure 10: L-Cry immunohistochemistry quantification

(a) Normalized CTCF values of cytoplasm and nucleus from independent individuals from four experimental repetitions. These values were used to calculate the nuclear/cytoplasmic ratio shown in Fig.6h. Biological replicates: NM zt0 -10min n=22; NM zt0 +10min n=18; FM7 zt0 -10min n=16**.** Individual data points as well as mean ± SEM are shown. **(b-u'')** Randomly selected, representative complete set of a L-Cry immunohistochemistry experimental repetition. Times as indicated, for further explanation see Fig.6a-a''.
