# Supplementary material for: A Cryptochrome adopts distinct moon- and sunlight states and functions as sun- versus moonlight interpreter in monthly oscillator entrainment
Source: Nat Commun. 2022 Sep 5;13:5220. doi: 10.1038/s41467-022-32562-z (PMC9445029; doi:10.1038/s41467-022-32562-z)
Supplement: Supplementary file 3 — Description of Additional Supplementary Files [file 41467_2022_32562_MOESM3_ESM.pdf]

Description of Additional Supplementary Files

File Name: Supplementary Data 1
Description: Raw data obtained from scoring L-Cry subcellular localization signal (see Supplementary Figure 8) using 40x confocal microscopy images like shown in the example in Suppl.Fig.8. (Excel table)
