# Supplementary material for: A Cryptochrome adopts distinct moon- and sunlight states and functions as sun- versus moonlight interpreter in monthly oscillator entrainment
Source: Nat Commun. 2022 Sep 5;13:5220. doi: 10.1038/s41467-022-32562-z (PMC9445029; doi:10.1038/s41467-022-32562-z)
Supplement: Supplementary file 5 — Reporting Summary [file 41467_2022_32562_MOESM5_ESM.pdf]

nature portfolio

Corresponding author(s): Eva Wolf
Kristin Tessmar-Raible

Last updated by author(s): 3.Juli 2022

Reporting Summary

Nature Portfolio wishes to improve the reproducibility of the work that we publish. This form provides structure for consistency and transparency in reporting. For further information on Nature Portfolio policies, see our Editorial Policies and the Editorial Policy Checklist.

Statistics

For all statistical analyses, confirm that the following items are present in the figure legend, table legend, main text, or Methods section.

n/a	Confirmed	
☐	☒	The exact sample size (*n*) for each experimental group/condition, given as a discrete number and unit of measurement
☐	☒	A statement on whether measurements were taken from distinct samples or whether the same sample was measured repeatedly
☐	☒	The statistical test(s) used AND whether they are one- or two-sided<br>*Only common tests should be described solely by name; describe more complex techniques in the Methods section.*
☐	☒	A description of all covariates tested
☐	☒	A description of any assumptions or corrections, such as tests of normality and adjustment for multiple comparisons
☐	☒	A full description of the statistical parameters including central tendency (e.g. means) or other basic estimates (e.g. regression coefficient) AND variation (e.g. standard deviation) or associated estimates of uncertainty (e.g. confidence intervals)
☐	☒	For null hypothesis testing, the test statistic (e.g. *F*, *t*, *r*) with confidence intervals, effect sizes, degrees of freedom and *P* value noted<br>*Give P values as exact values whenever suitable.*
☒	☐	For Bayesian analysis, information on the choice of priors and Markov chain Monte Carlo settings
☒	☐	For hierarchical and complex designs, identification of the appropriate level for tests and full reporting of outcomes
☒	☐	Estimates of effect sizes (e.g. Cohen's *d*, Pearson's *r*), indicating how they were calculated

Our web collection on statistics for biologists contains articles on many of the points above.

Software and code

Policy information about availability of computer code

Data collection	ChromLab V. 6.1., ASTRA 4.90.07, Agilent Open LAB CDS Chemstation, Spark Control, Zeiss ZEN 2012 SP5, Zeiss ZENPro 2 (blue edition), Image Lab Touch 3.0.1
Data analysis	R3.6.1, GraphPadPrism 8.4.2, Oriana 4.02, Microsoft Excel 2010, Origin version 7.5/10.5 (trial), ASTRA 4.90.07, Fiji/ImageJ 1.53c; Cellpose v0.6, Photoshop CC 2019 20.0

For manuscripts utilizing custom algorithms or software that are central to the research but not yet described in published literature, software must be made available to editors and reviewers. We strongly encourage code deposition in a community repository (e.g. GitHub). See the Nature Portfolio guidelines for submitting code & software for further information.

Data

Policy information about availability of data

All manuscripts must include a data availability statement. This statement should provide the following information, where applicable:

- Accession codes, unique identifiers, or web links for publicly available datasets
- A description of any restrictions on data availability
- For clinical datasets or third party data, please ensure that the statement adheres to our policy

Source data are provided with this paper in the Suppl. Figures and Suppl.Table 1. Full length l-cry cDNA- GenBank: MW161054. L-Cry fragment sequence for antibody production: GenBank ID: MT656570. All further source data can be downloaded from DRYAD: 10.5061/dryad.wm37pvmq8.

Field-specific reporting

Please select the one below that is the best fit for your research. If you are not sure, read the appropriate sections before making your selection.

☒ Life sciences ☐ Behavioural & social sciences ☐ Ecological, evolutionary & environmental sciences

For a reference copy of the document with all sections, see nature.com/documents/nr-reporting-summary-flat.pdf

Life sciences study design

All studies must disclose on these points even when the disclosure is negative.

Sample size

There was no predetermination of sample size. Where required for statistical analyses, sample size included at least three independent biological replicates. In many cases n is larger, depending on available material, how many samples can be processed reliable in parallel. As the data were highly reproducible, we concluded that the chosen sample size is sufficient to cover the biological variance. For long term spawning experiments, multiple biological replicates are impossible to collect for time and culture size reasons. (The study would probably take more then 5 years.) We based our judgement that the data are representative on at least two further re-entrainment experiments under artificial light that were shorter, but fully reproducible. The more precise spawning curves of l-cry-/- worms under artificial light reproduce very well as can be even seen in this manuscript with a different allele and under the re-entrainment conditions (before the re-entrainment starts). l-cry and r-opsin1 wildtype (and the indifferent r-opsin1-/- mutant) strain curves are very typical compared to other wildtype strains in the lab and thus representative for the normal spawning patterns of Platynereis under lab conditions. (Also see previous publications, e.g. Zantke et al. doi: 10.1534/genetics.112.148254, Zantke et al. doi: 10.1016/j.celrep.2013.08.031, Gühmann M, et al. doi: 10.1016/j.cub.2015.07.017.

Data exclusions

no data were excluded

Replication

For all spectroscopic experiments as well as for all SEC, SEC-MALS and HPLC analyses shown in Fig. 5, S5 and S6, at least two or three replicates were generated under each condition, which were consistent and confirmed reproducibility of the results. For dark recovery kinetics typically two to three replicates were done under each condition, which were consistent, i.e. confirmed reproducibility of the results. Several additional samplings of spawning data, as well as additional Western blots on L-Cry and immunohistochemical analyses for L-Cry localization confirm the presented data. Data were always consistent, i.e. confirmed reproducibility of the results (if everything was technically correctly performed under the correct conditions, e.g. no light pollution at night).

Randomization

Data analyses (and partially data collection) were blind and random for L-Cry subcellular localization and Western blots. As the data analyses were done blindly, there is no possibility to consciously or subconsciously influence the results in a specific direction.
For spawning data the knowledge of the different genotypes was (in most cases) present, but multiple people blind for the collected data of the others and the underlying experiment collectively collected the spawning data. The analyzing students then obtained lists of numbers that were combined according to genotype and/or condition for the calculations and analyzed. As the lists had to be combined, knowledge of genotype and/or condition was necessary at this moment, but as the numbers were already reported, there was no possibility that this could have influenced the analyses.
Randomization is not relevant for the UV/VIS, SEC, HPLC and SEC MALS experiments shown in Figures 5, S5 and S6, as these are automatized biophysical studies with well-defined parameters and statistics.

Blinding

Data analyses (and partially data collection) were blind and random for L-Cry subcellular localization and Western blots. As the data analyses were done blindly, there is no possibility to consciously or subconsciously influence the results in a specific direction.
For spawning data the knowledge of the different genotypes was (in most cases) present, but multiple people blind for the collected data of the others and the underlying experiment collectively collected the spawning data. The analyzing students then obtained lists of numbers that were combined according to genotype and/or condition for the calculations and analyzed. As the lists had to be combined, knowledge of genotype and/or condition was necessary at this moment, but as the numbers were already reported, there was no possibility that this could have influenced the analyses.
Blinding is not relevant for the UV/VIS, SEC, HPLC and SEC MALS experiments shown in Figures 5, S5 and S6, as these are automatized biophysical studies with well-defined parameters and statistics.

Behavioural & social sciences study design

All studies must disclose on these points even when the disclosure is negative.

Study description

Briefly describe the study type including whether data are quantitative, qualitative, or mixed-methods (e.g. qualitative cross-sectional, quantitative experimental, mixed-methods case study).

Research sample

State the research sample (e.g. Harvard university undergraduates, villagers in rural India) and provide relevant demographic information (e.g. age, sex) and indicate whether the sample is representative. Provide a rationale for the study sample chosen. For studies involving existing datasets, please describe the dataset and source.

Sampling strategy

Describe the sampling procedure (e.g. random, snowball, stratified, convenience). Describe the statistical methods that were used to predetermine sample size OR if no sample-size calculation was performed, describe how sample sizes were chosen and provide a rationale for why these sample sizes are sufficient. For qualitative data, please indicate whether data saturation was considered, and what criteria were used to decide that no further sampling was needed.

Data collection	*Provide details about the data collection procedure, including the instruments or devices used to record the data (e.g. pen and paper, computer, eye tracker, video or audio equipment) whether anyone was present besides the participant(s) and the researcher, and whether the researcher was blind to experimental condition and/or the study hypothesis during data collection.*
Timing	*Indicate the start and stop dates of data collection. If there is a gap between collection periods, state the dates for each sample cohort.*
Data exclusions	*If no data were excluded from the analyses, state so OR if data were excluded, provide the exact number of exclusions and the rationale behind them, indicating whether exclusion criteria were pre-established.*
Non-participation	*State how many participants dropped out/declined participation and the reason(s) given OR provide response rate OR state that no participants dropped out/declined participation.*
Randomization	*If participants were not allocated into experimental groups, state so OR describe how participants were allocated to groups, and if allocation was not random, describe how covariates were controlled.*

Ecological, evolutionary & environmental sciences study design

All studies must disclose on these points even when the disclosure is negative.

Study description	*Briefly describe the study. For quantitative data include treatment factors and interactions, design structure (e.g. factorial, nested, hierarchical), nature and number of experimental units and replicates.*
Research sample	*Describe the research sample (e.g. a group of tagged Passer domesticus, all Stenocereus thurberi within Organ Pipe Cactus National Monument), and provide a rationale for the sample choice. When relevant, describe the organism taxa, source, sex, age range and any manipulations. State what population the sample is meant to represent when applicable. For studies involving existing datasets, describe the data and its source.*
Sampling strategy	*Note the sampling procedure. Describe the statistical methods that were used to predetermine sample size OR if no sample-size calculation was performed, describe how sample sizes were chosen and provide a rationale for why these sample sizes are sufficient.*
Data collection	*Describe the data collection procedure, including who recorded the data and how.*
Timing and spatial scale	*Indicate the start and stop dates of data collection, noting the frequency and periodicity of sampling and providing a rationale for these choices. If there is a gap between collection periods, state the dates for each sample cohort. Specify the spatial scale from which the data are taken*
Data exclusions	*If no data were excluded from the analyses, state so OR if data were excluded, describe the exclusions and the rationale behind them, indicating whether exclusion criteria were pre-established.*
Reproducibility	*Describe the measures taken to verify the reproducibility of experimental findings. For each experiment, note whether any attempts to repeat the experiment failed OR state that all attempts to repeat the experiment were successful.*
Randomization	*Describe how samples/organisms/participants were allocated into groups. If allocation was not random, describe how covariates were controlled. If this is not relevant to your study, explain why.*
Blinding	*Describe the extent of blinding used during data acquisition and analysis. If blinding was not possible, describe why OR explain why blinding was not relevant to your study.*

Did the study involve field work? ☐ Yes ☒ No

Reporting for specific materials, systems and methods

We require information from authors about some types of materials, experimental systems and methods used in many studies. Here, indicate whether each material, system or method listed is relevant to your study. If you are not sure if a list item applies to your research, read the appropriate section before selecting a response.

Materials & experimental systems

n/a	Involved in the study	
☐	☒	Antibodies
☐	☒	Eukaryotic cell lines
☒	☐	Palaeontology and archaeology
☐	☒	Animals and other organisms
☒	☐	Human research participants
☒	☐	Clinical data
☒	☐	Dual use research of concern

Methods

n/a	Involved in the study	
☒	☐	ChIP-seq
☒	☐	Flow cytometry
☒	☐	MRI-based neuroimaging

Antibodies

Antibodies used	Anti-Actin, antibody produced in rabbit (Sigma, A2066, 048K4861); Anti-L-Cry, monoclonal produced in mouse (details in methods), secondary antibody Cy3 goat anti-mouse IgG (A10521, Thermo Fisher Scientific), secondary antibody anti-Mouse IgG-Peroxidase antibody, (Sigma, A4416, 1:7500); secondary antibody anti-rabbit IgG-HRP-linked antibody (Cell Signaling Technology, #7074, 1:7.500] , anti-Digoxigenin-AP coupled antibody Fab fragments (Roche Diagnostics, 11093274910), secondary antibody Alexa Fluor-488 goat anti-mouse IgG (Thermo Fisher Scientific), goat anti-rabbit IgG StarBright™ Blue 700 antibody (BIORAD)
Validation	Anti-Actin, see https://www.sigmaaldrich.com/AT/en/product/sigma/a2066; Anti-Lcry see Fig.1b-j for validation in western blot and immunohistochemistry<br>anti-Digoxigenin-AP coupled antibody Fab fragments are antibodies routinely used in RNA in situ hybridisations over the past >20 years.

Eukaryotic cell lines

Policy information about cell lines

Cell line source(s)	SF9 insect cells for Protein expression (#11496015, Thermo Fisher Scientific)
Authentication	no authentication
Mycoplasma contamination	not tested for Mycoplasma contamination
Commonly misidentified lines (See ICLAC register)	No commonly misidentified line was used in this study.

Palaeontology and Archaeology

Specimen provenance	*Provide provenance information for specimens and describe permits that were obtained for the work (including the name of the issuing authority, the date of issue, and any identifying information). Permits should encompass collection and, where applicable, export.*
Specimen deposition	*Indicate where the specimens have been deposited to permit free access by other researchers.*
Dating methods	*If new dates are provided, describe how they were obtained (e.g. collection, storage, sample pretreatment and measurement), where they were obtained (i.e. lab name), the calibration program and the protocol for quality assurance OR state that no new dates are provided.*

☐ Tick this box to confirm that the raw and calibrated dates are available in the paper or in Supplementary Information.

Ethics oversight	*Identify the organization(s) that approved or provided guidance on the study protocol, OR state that no ethical approval or guidance was required and explain why not.*

Note that full information on the approval of the study protocol must also be provided in the manuscript.

Animals and other organisms

Policy information about studies involving animals; ARRIVE guidelines recommended for reporting animal research

Laboratory animals	Platynereis dumerilii (invertebrate), Strains: Vio, Pin, L-cry mutants and wildtype of same origin (generated in Vio-background)<br>Sex: There are no distinct sex chromosomes in Platynereis (although sex determination is very likely genetic as the male/female ratio is typically 1.) Male versus female distinction can only be done based on morphological features.<br>Thus, in immature and premature stages the sexes were not discriminated. Experiments involving mature worms had about equal numbers of males and females.<br>Age: 3-18 months
Wild animals	No wild animals were used in this study.
Field-collected samples	We did not collect field samples for this study. Our work uses data from a field collection of worms in 1929 (published 1931), which is appropriately referred to and cited. (Ranzi, S. Ricerche sulla biologia sessuale degli Anellidi. Pubbl. Staz .Zool. Napoli 11, 271-292 (1931). Ranzi, S. Maturita sessuale degli Anellidi e fasi lunari. . Boll. Soc. Ital. Biol. Sperim. 6, 18 (1931).)
Ethics oversight	As these are invertebrates without special features grown in the lab for generations there are no ethical approvals required.

Note that full information on the approval of the study protocol must also be provided in the manuscript.

Human research participants

Policy information about studies involving human research participants

Population characteristics	*Describe the covariate-relevant population characteristics of the human research participants (e.g. age, gender, genotypic*

Population characteristics	*information, past and current diagnosis and treatment categories). If you filled out the behavioural & social sciences study design questions and have nothing to add here, write "See above."*
Recruitment	*Describe how participants were recruited. Outline any potential self-selection bias or other biases that may be present and how these are likely to impact results.*
Ethics oversight	*Identify the organization(s) that approved the study protocol.*

Note that full information on the approval of the study protocol must also be provided in the manuscript.

Clinical data

Policy information about clinical studies

All manuscripts should comply with the ICMJE guidelines for publication of clinical research and a completed CONSORT checklist must be included with all submissions.

Clinical trial registration	*Provide the trial registration number from ClinicalTrials.gov or an equivalent agency.*
Study protocol	*Note where the full trial protocol can be accessed OR if not available, explain why.*
Data collection	*Describe the settings and locales of data collection, noting the time periods of recruitment and data collection.*
Outcomes	*Describe how you pre-defined primary and secondary outcome measures and how you assessed these measures.*

Dual use research of concern

Policy information about dual use research of concern

Hazards

Could the accidental, deliberate or reckless misuse of agents or technologies generated in the work, or the application of information presented in the manuscript, pose a threat to:

No	Yes	
☒	☐	Public health
☒	☐	National security
☒	☐	Crops and/or livestock
☒	☐	Ecosystems
☒	☐	Any other significant area

Experiments of concern

Does the work involve any of these experiments of concern:

No	Yes	
☒	☐	Demonstrate how to render a vaccine ineffective
☒	☐	Confer resistance to therapeutically useful antibiotics or antiviral agents
☒	☐	Enhance the virulence of a pathogen or render a nonpathogen virulent
☒	☐	Increase transmissibility of a pathogen
☒	☐	Alter the host range of a pathogen
☒	☐	Enable evasion of diagnostic/detection modalities
☒	☐	Enable the weaponization of a biological agent or toxin
☒	☐	Any other potentially harmful combination of experiments and agents

ChIP-seq

Data deposition

☐ Confirm that both raw and final processed data have been deposited in a public database such as GEO.

☐ Confirm that you have deposited or provided access to graph files (e.g. BED files) for the called peaks.

Data access links<br>*May remain private before publication.*	*For "Initial submission" or "Revised version" documents, provide reviewer access links. For your "Final submission" document, provide a link to the deposited data.*
Files in database submission	*Provide a list of all files available in the database submission.*

Genome browser session
(e.g. UCSC)

Provide a link to an anonymized genome browser session for "Initial submission" and "Revised version" documents only, to enable peer review. Write "no longer applicable" for "Final submission" documents.

Methodology

Replicates

Describe the experimental replicates, specifying number, type and replicate agreement.

Sequencing depth

Describe the sequencing depth for each experiment, providing the total number of reads, uniquely mapped reads, length of reads and whether they were paired- or single-end.

Antibodies

Describe the antibodies used for the ChIP-seq experiments; as applicable, provide supplier name, catalog number, clone name, and lot number.

Peak calling parameters

Specify the command line program and parameters used for read mapping and peak calling, including the ChIP, control and index files used.

Data quality

Describe the methods used to ensure data quality in full detail, including how many peaks are at FDR 5% and above 5-fold enrichment.

Software

Describe the software used to collect and analyze the ChIP-seq data. For custom code that has been deposited into a community repository, provide accession details.

Flow Cytometry

Plots

Confirm that:

☐ The axis labels state the marker and fluorochrome used (e.g. CD4-FITC).

☐ The axis scales are clearly visible. Include numbers along axes only for bottom left plot of group (a 'group' is an analysis of identical markers).

☐ All plots are contour plots with outliers or pseudocolor plots.

☐ A numerical value for number of cells or percentage (with statistics) is provided.

Methodology

Sample preparation

Describe the sample preparation, detailing the biological source of the cells and any tissue processing steps used.

Instrument

Identify the instrument used for data collection, specifying make and model number.

Software

Describe the software used to collect and analyze the flow cytometry data. For custom code that has been deposited into a community repository, provide accession details.

Cell population abundance

Describe the abundance of the relevant cell populations within post-sort fractions, providing details on the purity of the samples and how it was determined.

Gating strategy

Describe the gating strategy used for all relevant experiments, specifying the preliminary FSC/SSC gates of the starting cell population, indicating where boundaries between "positive" and "negative" staining cell populations are defined.

☐ Tick this box to confirm that a figure exemplifying the gating strategy is provided in the Supplementary Information.

Magnetic resonance imaging

Experimental design

Design type

Indicate task or resting state; event-related or block design.

Design specifications

Specify the number of blocks, trials or experimental units per session and/or subject, and specify the length of each trial or block (if trials are blocked) and interval between trials.

Behavioral performance measures

State number and/or type of variables recorded (e.g. correct button press, response time) and what statistics were used to establish that the subjects were performing the task as expected (e.g. mean, range, and/or standard deviation across subjects).

Acquisition

Imaging type(s) — *Specify: functional, structural, diffusion, perfusion.*

Field strength — *Specify in Tesla*

Sequence & imaging parameters — *Specify the pulse sequence type (gradient echo, spin echo, etc.), imaging type (EPI, spiral, etc.), field of view, matrix size, slice thickness, orientation and TE/TR/flip angle.*

Area of acquisition — *State whether a whole brain scan was used OR define the area of acquisition, describing how the region was determined.*

Diffusion MRI ☐ Used ☐ Not used

Preprocessing

Preprocessing software — *Provide detail on software version and revision number and on specific parameters (model/functions, brain extraction, segmentation, smoothing kernel size, etc.).*

Normalization — *If data were normalized/standardized, describe the approach(es): specify linear or non-linear and define image types used for transformation OR indicate that data were not normalized and explain rationale for lack of normalization.*

Normalization template — *Describe the template used for normalization/transformation, specifying subject space or group standardized space (e.g. original Talairach, MNI305, ICBM152) OR indicate that the data were not normalized.*

Noise and artifact removal — *Describe your procedure(s) for artifact and structured noise removal, specifying motion parameters, tissue signals and physiological signals (heart rate, respiration).*

Volume censoring — *Define your software and/or method and criteria for volume censoring, and state the extent of such censoring.*

Statistical modeling & inference

Model type and settings — *Specify type (mass univariate, multivariate, RSA, predictive, etc.) and describe essential details of the model at the first and second levels (e.g. fixed, random or mixed effects; drift or auto-correlation).*

Effect(s) tested — *Define precise effect in terms of the task or stimulus conditions instead of psychological concepts and indicate whether ANOVA or factorial designs were used.*

Specify type of analysis: ☐ Whole brain ☐ ROI-based ☐ Both

Statistic type for inference (See Eklund et al. 2016) — *Specify voxel-wise or cluster-wise and report all relevant parameters for cluster-wise methods.*

Correction — *Describe the type of correction and how it is obtained for multiple comparisons (e.g. FWE, FDR, permutation or Monte Carlo).*

Models & analysis

n/a	Involved in the study
☐	☐ Functional and/or effective connectivity
☐	☐ Graph analysis
☐	☐ Multivariate modeling or predictive analysis

Functional and/or effective connectivity — *Report the measures of dependence used and the model details (e.g. Pearson correlation, partial correlation, mutual information).*

Graph analysis — *Report the dependent variable and connectivity measure, specifying weighted graph or binarized graph, subject- or group-level, and the global and/or node summaries used (e.g. clustering coefficient, efficiency, etc.).*

Multivariate modeling and predictive analysis — *Specify independent variables, features extraction and dimension reduction, model, training and evaluation metrics.*
