# Supplementary figures and images for: A Cryptochrome adopts distinct moon- and sunlight states and functions as sun- versus moonlight interpreter in monthly oscillator entrainment
Source: Nat Commun. 2022 Sep 5;13:5220. doi: 10.1038/s41467-022-32562-z (PMC9445029; doi:10.1038/s41467-022-32562-z)

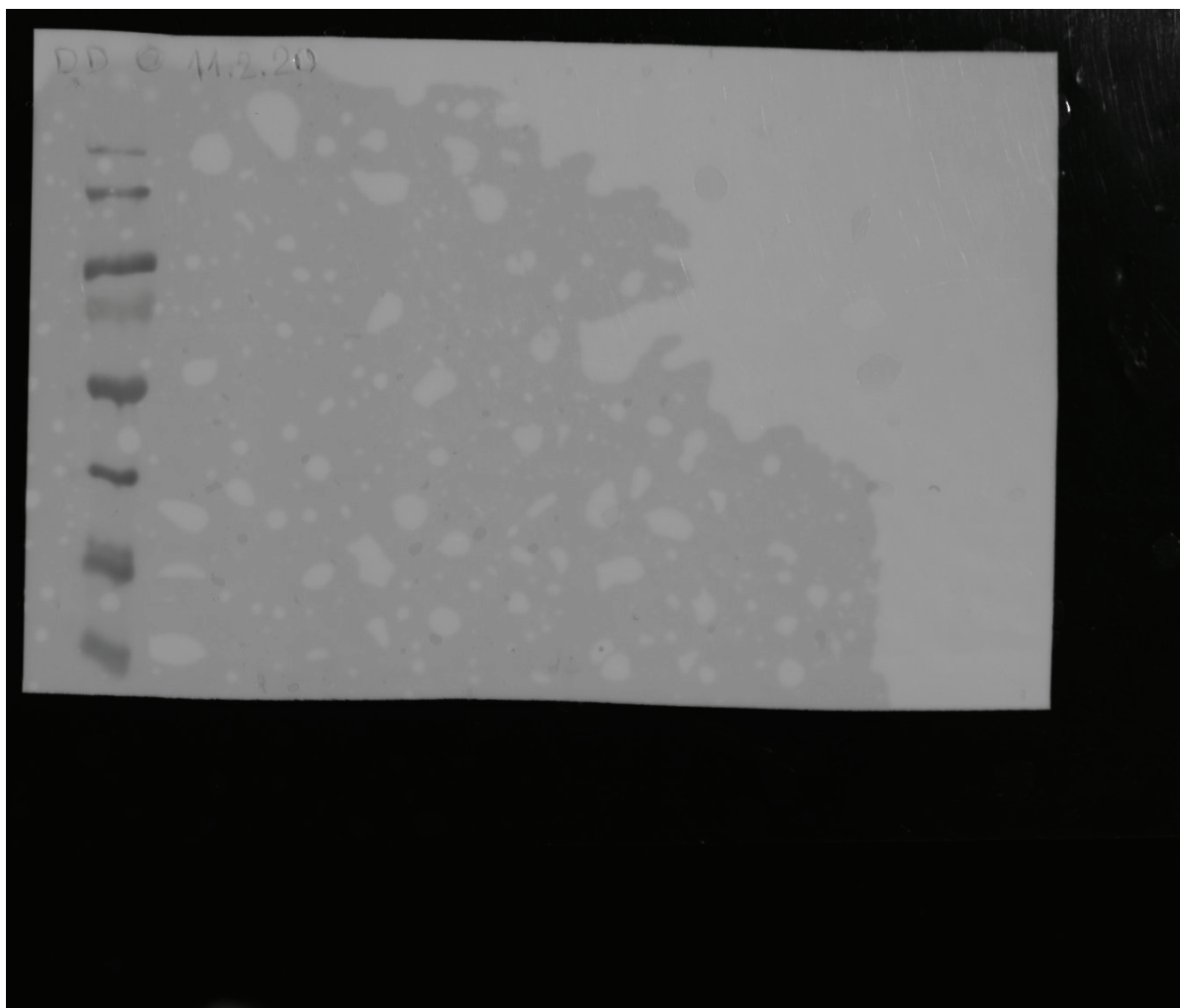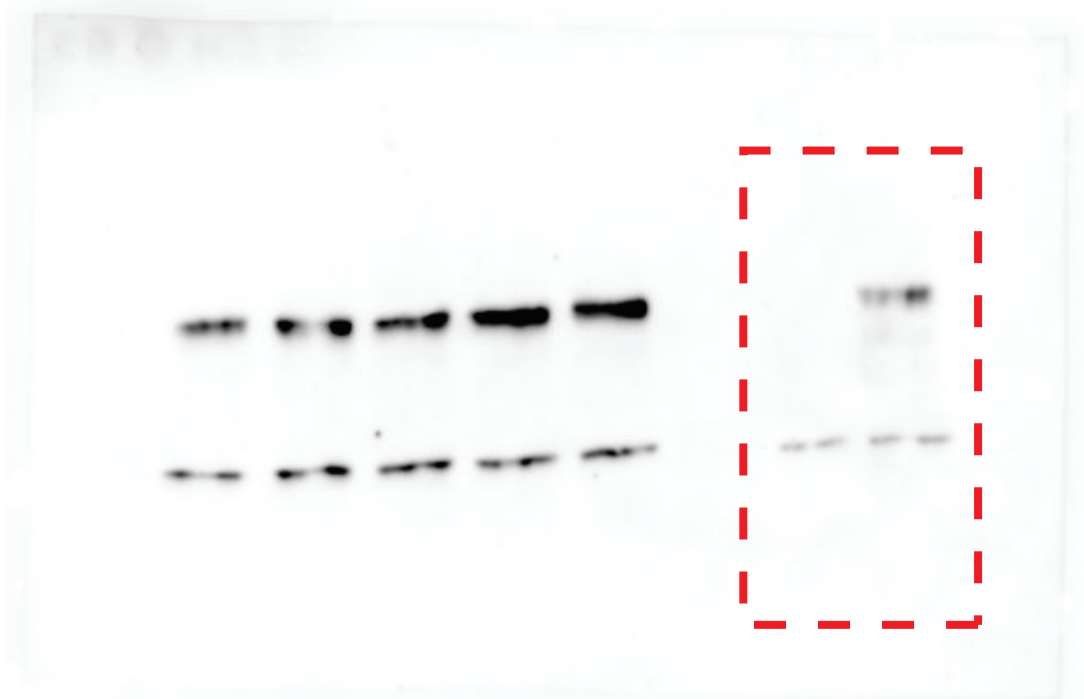

Supplement: Supplementary file 6 — Source Data [file 41467_2022_32562_MOESM6_ESM.zip › Source_Data_PoehnKrishnanetal/Poehn_Krishnan_2022_Fig.1b_right.pdf]

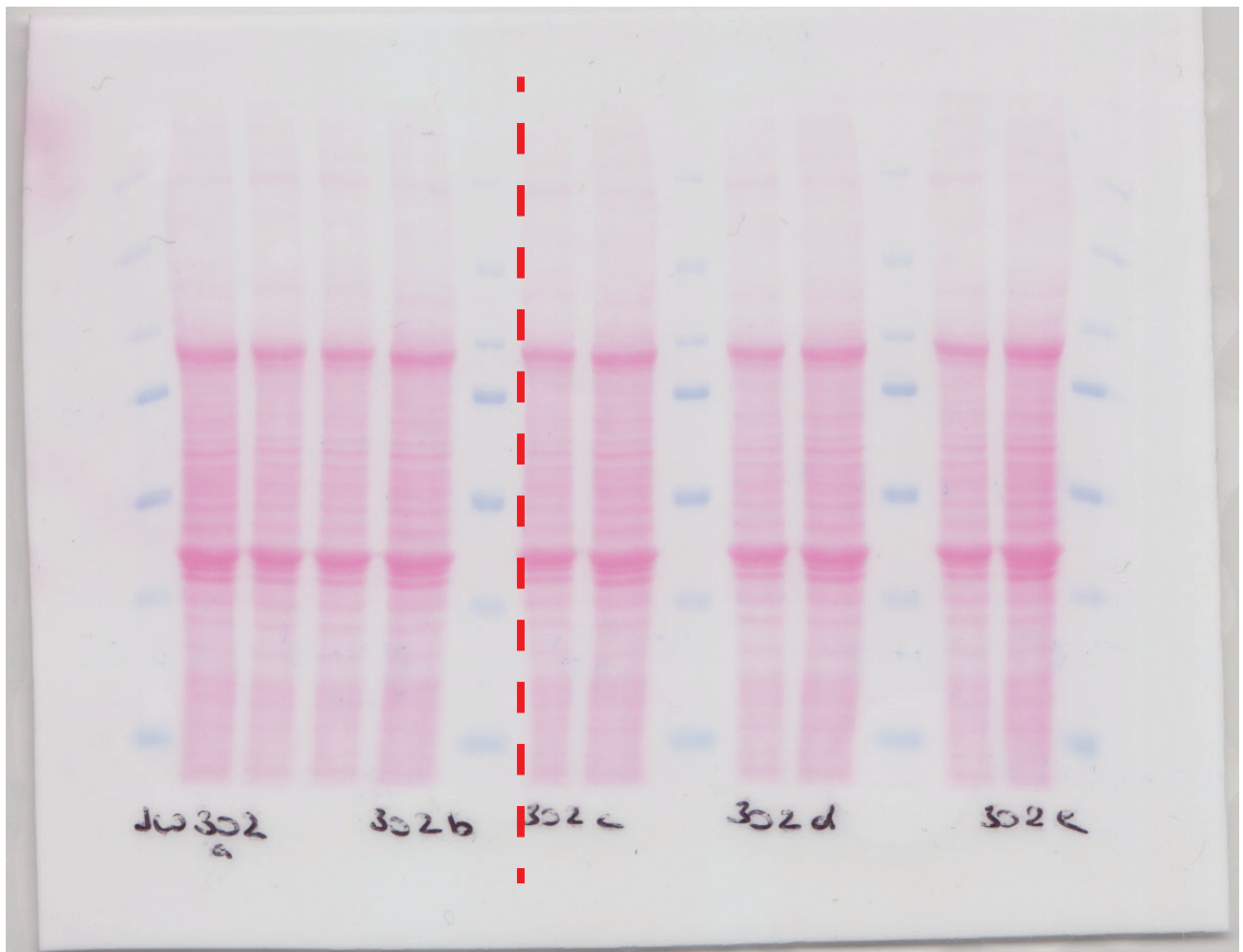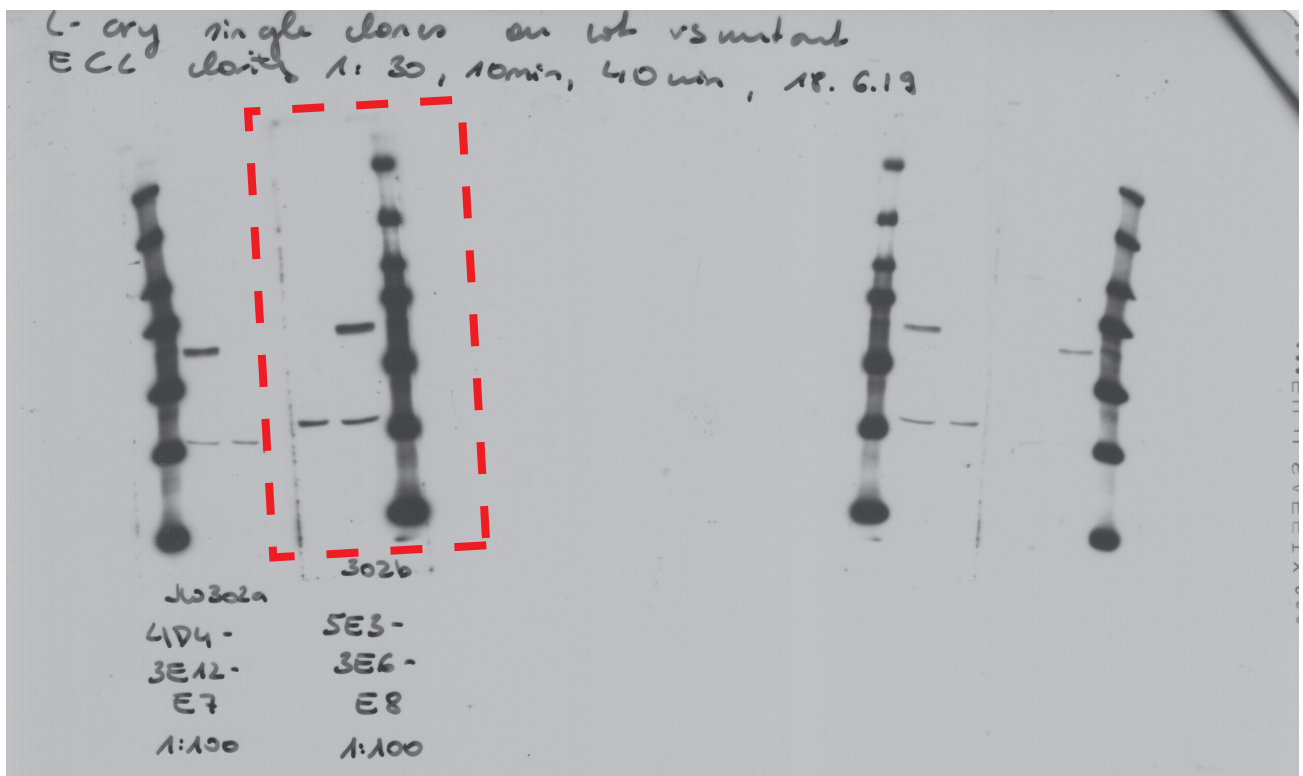

Supplement: Supplementary file 6 — Source Data [file 41467_2022_32562_MOESM6_ESM.zip › Source_Data_PoehnKrishnanetal/Poehn_Krishnan_2022_Fig.1b_left.pdf]

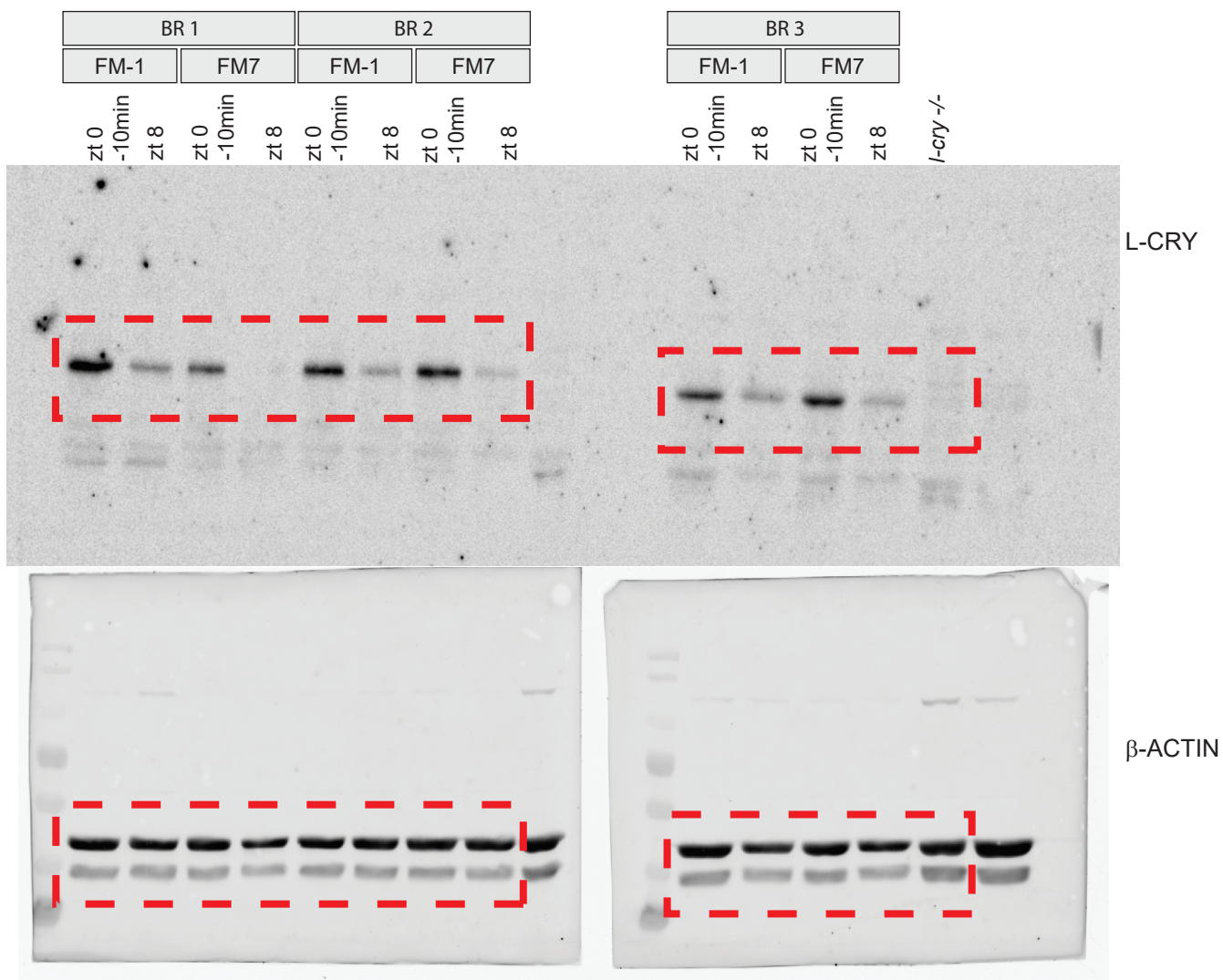

Supplementary Figure 7 part 1

Supplement: Supplementary file 6 — Source Data [file 41467_2022_32562_MOESM6_ESM.zip › Source_Data_PoehnKrishnanetal/Poehn_Krishnan_2022_Fig6c_Suppl.fig.7_page1.pdf]

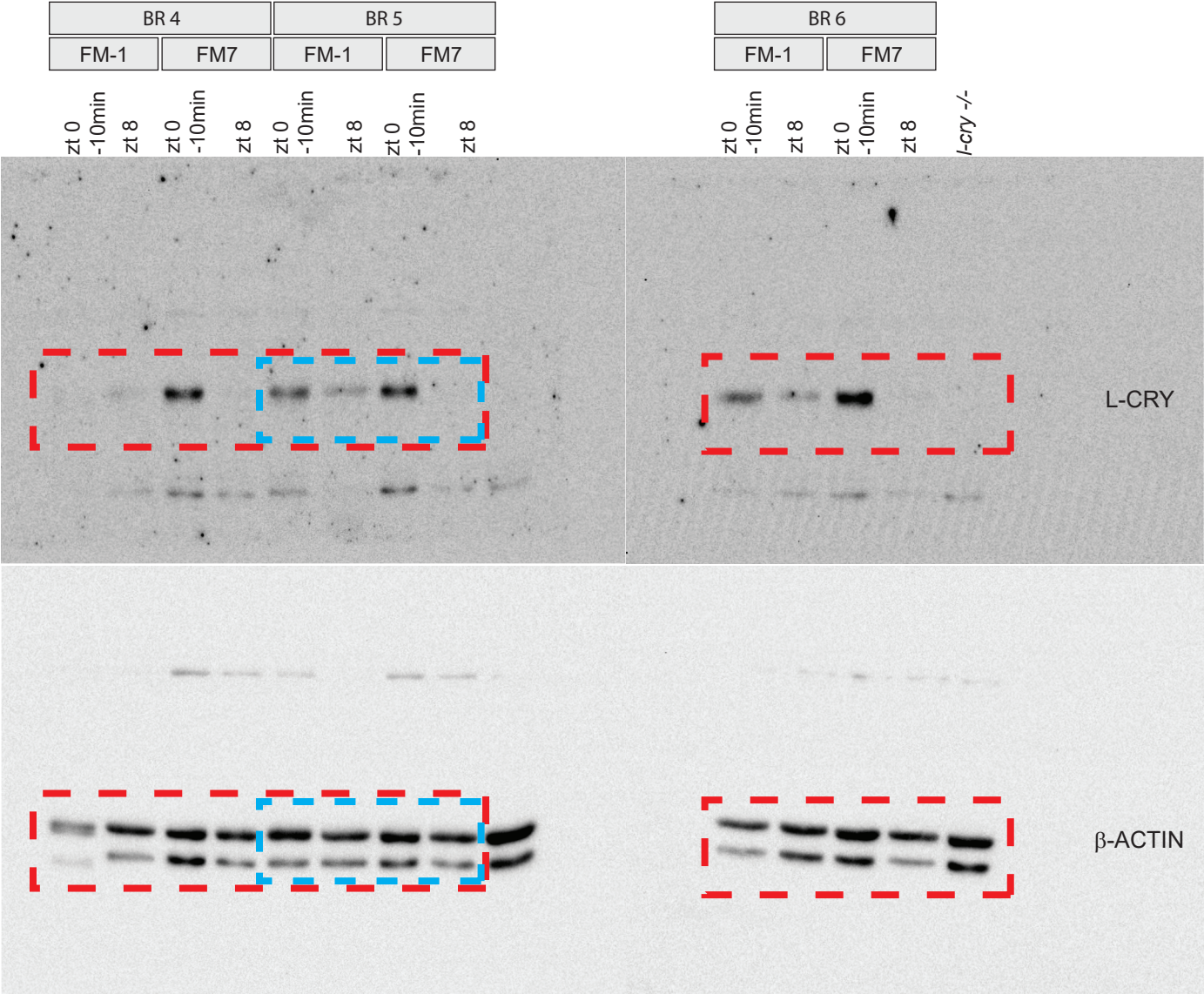

Supplementary Figure 7 part 2

Supplement: Supplementary file 6 — Source Data [file 41467_2022_32562_MOESM6_ESM.zip › Source_Data_PoehnKrishnanetal/Poehn_Krishnan_2022_Fig6c_Suppl.fig.7_page2.pdf]

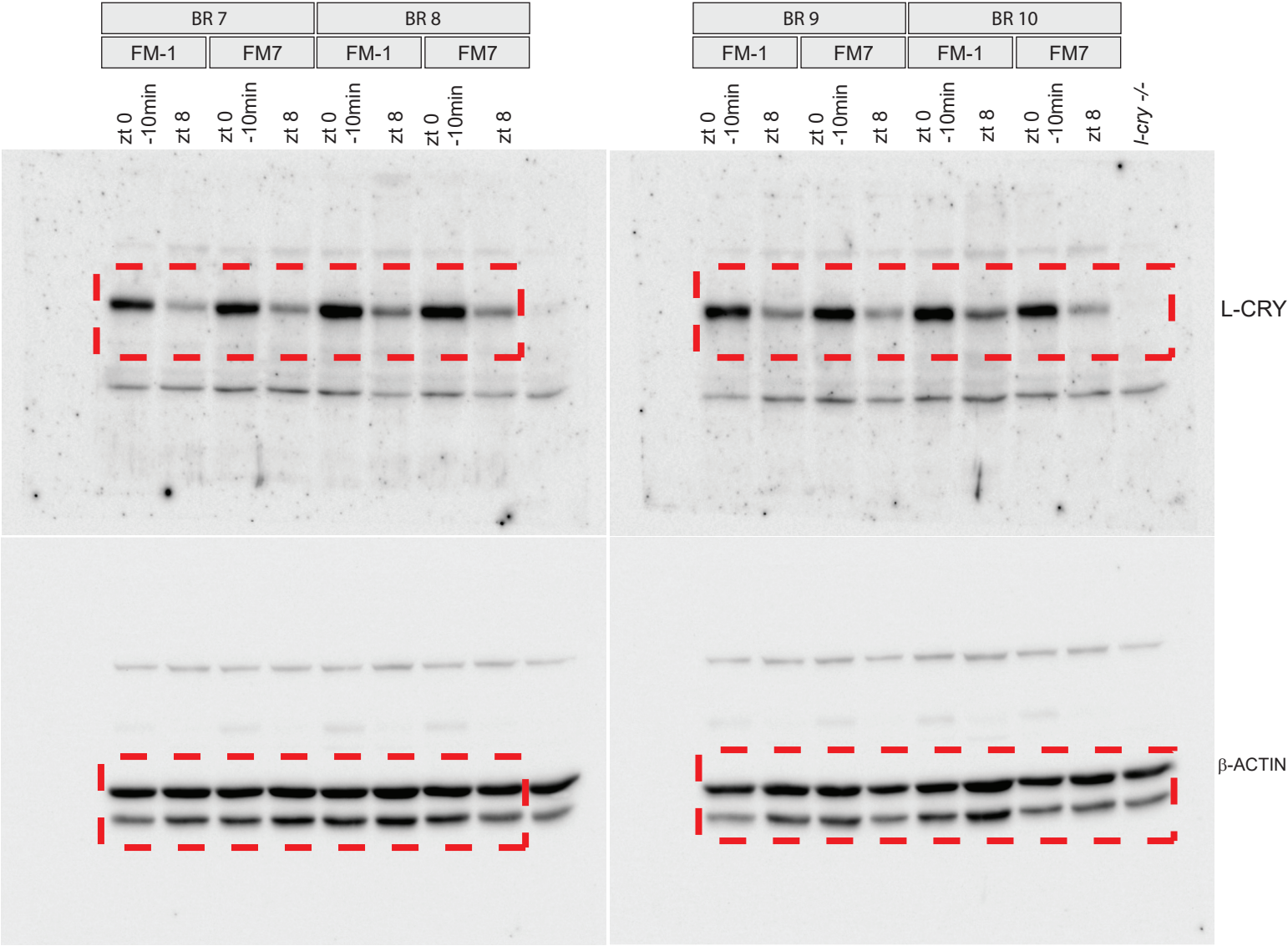

Supplementary Data Figure 7 part 3

Supplement: Supplementary file 6 — Source Data [file 41467_2022_32562_MOESM6_ESM.zip › Source_Data_PoehnKrishnanetal/Poehn_Krishnan_2022_Fig6c_Suppl.fig.7_page3.pdf]
